# Metabolome analysis and chemical profiling of Indonesian royal jellies as the raw material for cosmetic and bio-supplement products

**DOI:** 10.1016/j.heliyon.2021.e06912

**Published:** 2021-05-03

**Authors:** Eka Sari, Kaysa Faradis Mahira, Dhavalkumar Narendrabhai Patel, Lee Suan Chua, Diah Kartika Pratami, Muhamad Sahlan

**Affiliations:** a Bioengineering and Biomedical Engineering Laboratory, Research Centre Sultan Ageng Tirtayasa, Banten, 42124, Indonesia; b Chemical Engineering, Faculty of Engineering, Universitas Sultan Ageng Tirtayasa, Banten, 42124, Indonesia; c Department of Chemical Engineering, Faculty of Engineering, Universitas Indonesia, Kampus UI, Depok, 16424, Indonesia; d Waters Pacific Pte Ltd, Singapore, 117528, Singapore; e Institute of Bioproduct Development, Universiti Teknologi Malaysia, 81310, Johor Bahru, Malaysia; f Laboratorium of Pharmacognosy and Phytochemistry, Faculty of Pharmacy, Pancasila University, Jakarta, 12640, Indonesia

**Keywords:** Royal jelly, Sebacic acid, Skin, Cosmetic, Metabolomics

## Abstract

Royal jellies (RJs) possess moisturizing, emulsifying, and stabilizing properties, and several pharmacological activities have also been found to be present, which make them an ideal component for cosmetic and skin care products. However, despite the abundant efficacies, there is a lack of studies that explore the chemical composition of RJ using metabolome analysis. Furthermore, an evaluation of the chemical composition of Indonesian RJs collected from different regions has yet to be carried out. Therefore, the main objective of this study was to identify any differences in the chemical composition of such RJs. Chemical profiling was also carried out to enable more targeted utilization based on the actual compositions. Chemical profiling is also important given the rich Indonesian biodiversity and the high dependence of the RJ compositions on the botanical source. In this research, ultra-performance liquid chromatography coupled with quadrupole time-of-flight mass spectrometry was used as part of an untargeted metabolomics approach. From the chemical profiling, >30 compounds were identified across four RJ samples. The major constituents of the samples were found to be oligosaccharides, fatty acids, and adenosine monophosphate derivatives. Meanwhile, sucrose and planteose were found to be highest in the samples from Banjarnegara and Kediri, whereas dimethyloctanoic acid was found to be unique to the sample from Banjarnegara. It was also discovered that the RJs from Demak and Tuban contained more organic fatty acids and oligosaccharides than the other samples. Although the sample from Demak demonstrated good potential for use in the cosmetic, skin care, and bio-supplement industries, the higher abundance of fatty acids and oligosaccharides in the sample from Tuban indicated that it is perhaps the most suitable RJ for use in this field.

## 1 Introduction

Royal jelly (RJ), or bee's milk, is a yellowish creamy glandular secretion with a gelatinous–viscous texture, unique phenol smell, and sour taste (Fratini et al., 2016). Secreted by the young nurse worker bees through hypopharyngeal and mandibular glands, RJ is consumed by larvae and adult queens to meet their nutritional needs (Patel et al. 1960; Morita et al., 2012). The nutritious RJ is the main reason for the longer life of queen bee over other bees (Pavel et al., 2011).

RJ is reported to be composed of water (60%–70%), proteins (27%–41% of the dry matter), carbohydrates (up to 30% of the dry matter), fatty acids and lipids (3%–8% of the fresh matter and 8%–19% of the lyophilized form), 10-hydroxy-2-decenoic acid (10-HDA) (>1.4% of the fresh matter and >3.5% of the lyophilized form), and trace amounts of vitamins and minerals (Sabatini et al., 2009). Because of the high abundance of organic fatty acids and oligosaccharides, RJs possess moisturizing, emulsifying, and stabilizing properties, and several pharmacological activities are also present, including antioxidative, antimicrobial, anti-inflammatory, antitumor, anti-hypertensive, anti-photoaging, and wound-healing properties (Pavel et al., 2011; Townsend et al., 1960; Tokunaga et al., 2004; Park et al. 2011, 2012), which make them an ideal constituent for cosmetic and skin care products.

Unlike other hive products such as honey, bee pollen, and propolis, RJ has not been widely studied. The scientific data on the use and biochemistry of RJ are also considered to be insufficient for supporting all the reported RJ effects and efficacies (Pavel et al., 2011). In fact, although in the last two decades there has been a growing interest in RJ research globally, a metabolomics-based evaluation of the chemical composition of RJ collected from different regions has yet to be carried out. Indonesia has various types of RJ-producing bees that inhabit different areas, and the chemical profiling of Indonesian RJ from different regions is crucial given the rich Indonesian biodiversity and the high dependence of the RJ compositions on the botanical source. This research could provide a number of benefits, including increasing the utilization of Indonesian RJs in producing cosmetic, skin care, and bio-supplement products.

In this research, untargeted chemical profiling is used to measure as many metabolites in the samples as possible and to obtain complete chemical signatures for comparison. Chemical profiling through multivariate modeling and chemometrics enables researchers to detect key marker compounds that are unique to a specific sample, as well as its properties. Among the various available approaches, liquid chromatography (LC)–mass spectrometry (MS) is a widely used analytical technique with high separation and detection sensitivity to diverse chemical components. Meanwhile, methods based on rapid chromatographic separation and high-resolution MS are being increasingly employed and ultra-performance LC (UPLC) has become a prevalent analytical platform in chemical profiling filed as it employs higher pressure of 12,000–15,000 p.s.i compared to high-performance LC and generates smaller particle size of approximately 2 μm which accommodates more rapid and efficient separation for complex natural product mixtures (Yuk et al., 2016).

The objective of this study is to identify the differences in the chemical compositions of various RJs obtained from different locations in Indonesia through chemical profiling and multivariate analysis. In addition, each detected chemical is checked for its potential use in cosmetic, skin care, and bio-supplement products to ensure more targeted utilization based on the actual compositions. Recommendations for RJ utilization are provided in view of the comparison of the chemical data for each sample in relation to the existing research related to skin care and supplement products. Four types of Indonesian RJ from Tuban, Kediri, Demak, and Banjarnegara are analyzed using UPLC coupled with a quadrupole time-of-flight MS (QTOF/MS) as part of an untargeted metabolomics approach.

## 2 Materials and methods

**Figure 1:**
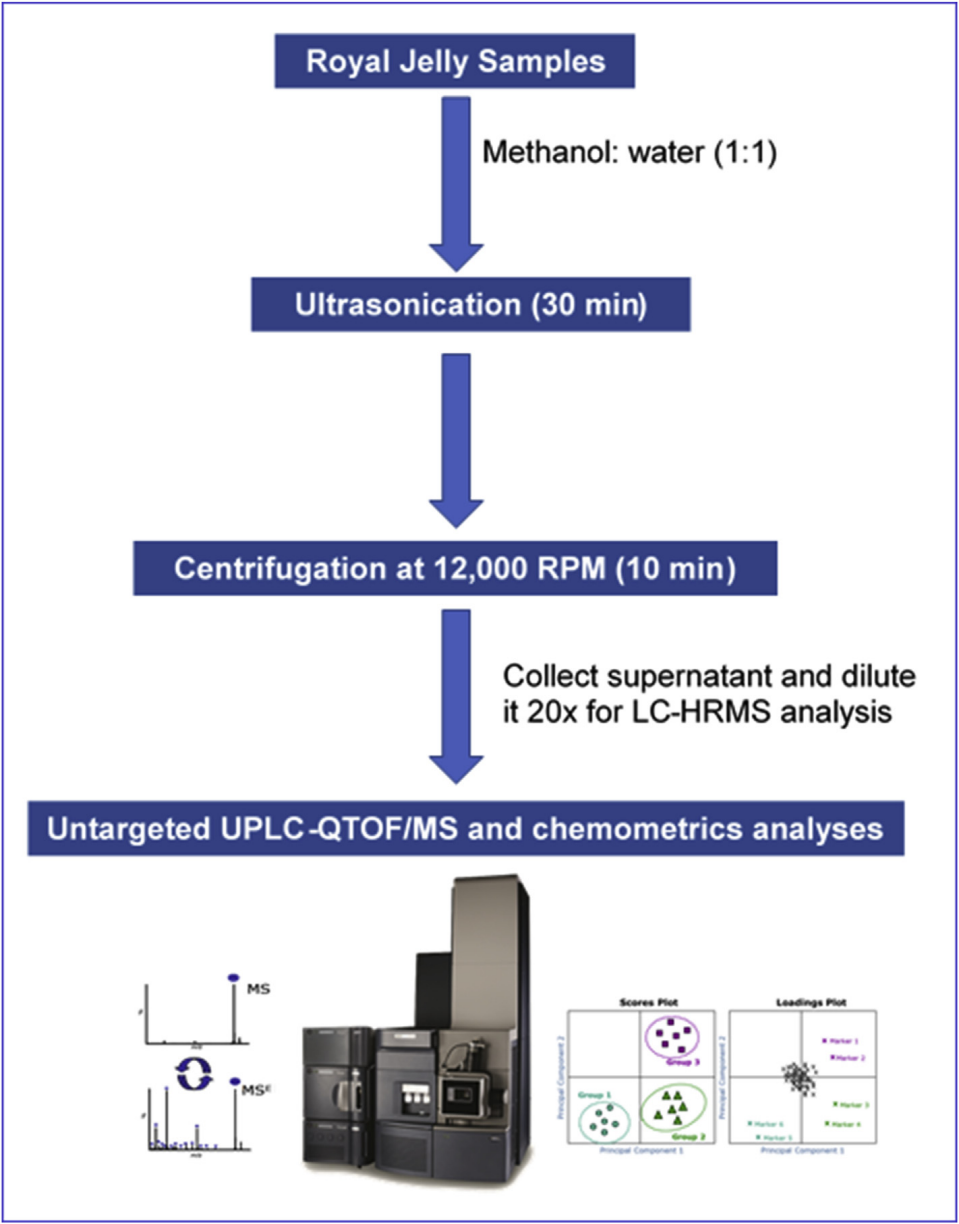
Flow chart of overall methodology.

The methods were divided into three main parts, which were sample preparation, UPLC-QTOF/MS analysis and data analysis. The workflow is available in Figure 1.

### 2.1 Sample preparation

Different types of RJ from four locations in Indonesia, Tuban, Kediri, Demak, and Banjarnegara, were obtained and labeled as T, K, M, and B, respectively. All samples were extracted in triplicate with 1:1 methanol: water (LC–MS grade). The samples were vortexed for 1 min following the addition of the solvent before sonication was performed for 30 min. The samples were centrifuged for 10 min at 12,000 rpm subsequent to the extraction. Post centrifugation, the supernatants were collected and diluted 20 times for LC–MS injection.

### 2.2 UPLC–QTOF/MS data acquisition

Untargeted chemical profiling and metabolite analysis was performed on ACQUITY I-Class UPLC coupled to Xevo G2-XS QTOF system (Waters Corporation, Milford, USA). Here, a reverse-phase HSS T3 C18 column (2.1 mm × 150 mm, I.D. 1.8 μm) was utilized and maintained at 40 °C. Two types of mobile phases were used which were A (water in 0.1 % formic acid) and B (acetonitrile in 0.1 % formic acid) phases. The methods for operating the UPLC was adapted from Yuk et al. (2016) study with some modifications. Gradient elution was performed at a flow rate of 0.45 mL min^−1^ with an injection volume of 1 μL using following gradient program: 0.5 % B (0–2 min), 0.5–20 % B (2–4 min), 50 % B (4–7 min), 50–99 % B (7–11 min), 99 % B (11–13 min), and 2-min equilibration to 0.5 % B (13–15 min).

Accurate mass data were acquired by using QTOF mass spectrometer controlled by UNIFI 1.8.3 informatics platform (Waters Corporation, Milford, USA) in both negative and positive electrospray ionization (ESI) modes over a mass range of 50 to 1,200 Da in a data independent acquisition mode (MS^E^ mode). This acquisition mode allows simultaneous collection of full scan MS data at low and high collision energies to obtain comprehensive information on precursor ions and corresponding daughter or fragment ions of all ionizable species of a chromatographic separation in a single injection. Such DIA data also facilitates confirmation of potential hits obtained from database searches without reanalyzing sample using MS fragmentation data collected from high energy scans. Leucine enkephalin (200 pg μL^−1^) acted as the lock mass compound (reference compound) with *m/z* of 556.2766. The source temperature and desolvation gas flows was set at 120 °C and 900 L h^−1^ at 550 °C respectively. The capillary voltage was set to −1.5 kV and the cone voltage to 40 V. The low-energy scan was set at 6 eV whilst the high-energy scan was set at a ramp energy scan of 10–45 eV. Both functions have scan time of 0.10 s. From the initial data evaluation, ESI negative mode yielded more compound coverage and hence it was used for the metabolomics experiments. There were total of three injections undertook for each sample including the pooled sample (used as the quality-control sample) which were then randomized prior to the data acquisition.

### 2.3 Data and statistical analysis

UNIFI software was used for processing, peak picking, and analyzing all of the acquired LC-MS data. The peak apexes of all ion responses were detected using a 3D peak detection algorithm based on their 3D shapes, which allowed us to obtain cleaner spectra and more accurate peak volumes than with 2D-extracted ion chromatograms. According to Yuk et al. (2016) method, in order to generate a matrix consisting of the *m/z* value, retention time (RT), and normalized peak area, the total intensity of each ion was normalized to the total ion count. The acquired data matrix was used in the multivariate statistical analysis using EZinfo 3.03 software (Umetrics, Umea, Sweden). For principal component analysis (PCA) purposes, the data were mean-centered and center-scaled. The variables of interest were subsequently identified using the discovery tool in the UNIFI software, which connects directly to an in-house traditional medicine library.

## 3 Results and discussion

### 3.1 Principal component analysis

**Figure 2:**
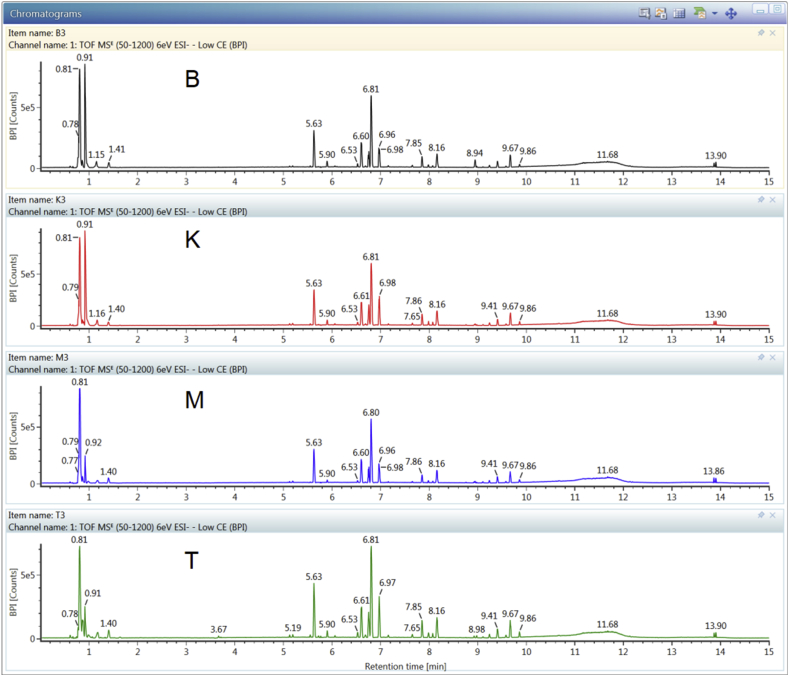
UPLC-QTOF/MS BPI chromatograms of four RJ samples from Banjarnegara (B), Kediri (K), Demak (M), and Tuban (T) in Indonesia.

Figure 2 reveals the LC–MS chromatograms of all RJ samples. Many of the peaks were well separated in a 15–min run using one solvent extraction with methanol: water (1:1) for all samples combined with an optimal UPLC method. However, it was rather difficult to observe the variation in their chromatograms using a visual comparison.

**Figure 3:**
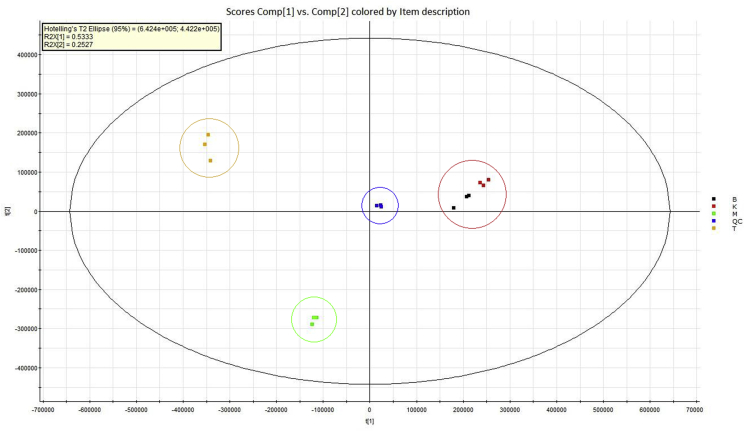
The PCA scores plot for the RJ samples. The first two principal components are shown. The ellipse represents the 95% confidence interval of the PCA model. Black: Banjarnegara (B), Red: Kediri (K), Green: Demak (M), and Orange: Tuban (T).

Multivariate statistical modeling via PCA was subsequently adopted to fully understand the similarities or differences among the samples and also to identify the target chemical markers that differentiate them. The exemption of class discrimination during the calculation and the peculiar clustering and appealing deviation from complex datasets it resulted have made PCA considered to be equitable tools (Nicholson et al., 2007). In order to cluster the RJ samples, a PCA scores plot was constructed as shown in Figure 3.

**Figure 4:**
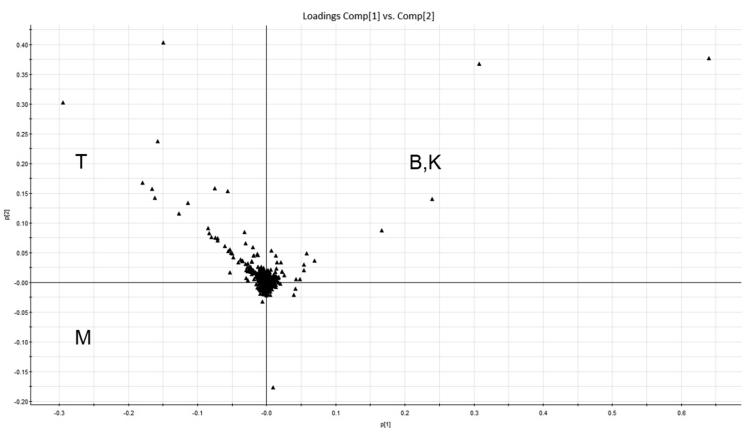
The PCA loadings plot for the four RJ samples with each dot representing a *m/z*–RT variable. The selected variables (red box) are shown as compounds unique to sample T.

As presented in Figure 3, in PCA score plot, a particular symbol is used to represent each sample. In addition, the plot distances from each other really matter as they reveals how closely related the chemical profiles each sample has from one another. Peak number (*m/z*–RT pair) and the normalized peak areas were utilized as the variables for the assessment. From the scores plot in Figure 3, it is clear that the observed variables in the T and M samples were very different to the other samples, whereas the B and K samples appeared to have few differences. There was also an excellent reproducibility for all samples with strict grouping of replicates and also clustering of quality control samples at the center of the PCA score plot. From these result, the first two principal components were used, which accounted for 78.6% of the total variance described. To better understand the specific chemical variations causing the samples' profile differences in PCA scores, a loadings plot was generated as shown in Figure 4.

Each point on the loadings plot (Figure 4) represents the *m/z*–RT pair which complements the information in the score plot by displaying variables that are important in distinguishing sample. There were three distinct quadrants that classify each RJ sample. The RJ samples in the scores plot have many variables (*m/z*) associated in that area. The points in the center of the plot were other variables that were considered not to be significantly different across all the samples and hence would be excluded in further analysis.

### 3.2 Chemical profiling for chemical and marker identification

The LC–MS/MS results only provided spectrum data, and it proved difficult to identify each chemical component through visual screening alone. Hence, chemical profiling using UNIFI software was carried out to provide clearer explanations. The UNIFI software also allowed us to further comprehend the chemical profile differences in terms of tagging and linking the key differentiating variables (*m/z*–RT pairs) in each of the quadrants into the UNIFI discovery tool connected to the in-house traditional medicine library. With reference to the medicine library, which consists of 600 herbs and ~6,400 compounds, the variables were screened for any potential markers.

**Figure 5:**
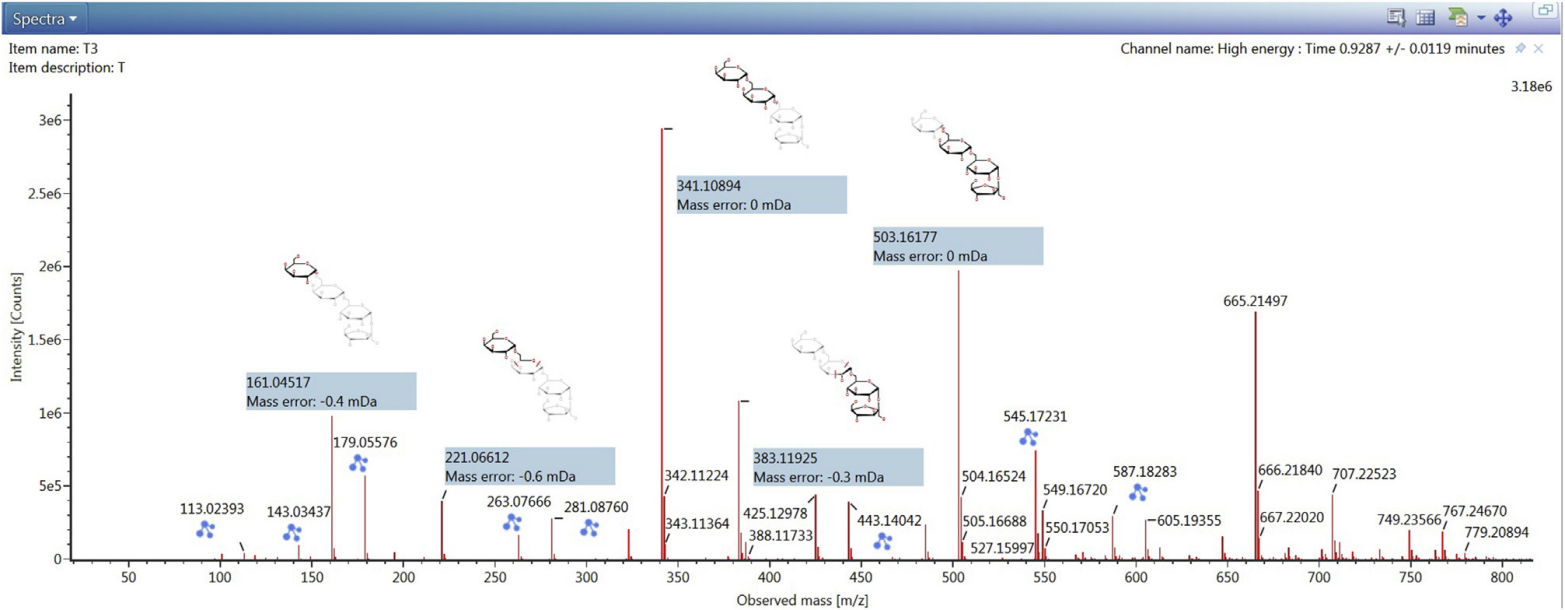
MSMS spectrum of stachyose, identified as one of the major marker constituents of royal jelly sample collected from Tuban (T), Indonesia.

The confirmation of parent and fragment ions from the loadings plot (Figure 4) generated a higher confidence for marker identification. The main compounds that distinguished the four RJ samples were successfully identified. The component names, relative abundances, and standard deviations are listed in Table 1. The utilization of UPLC-QTOF/MS with a streamlined informatics workflow was fast and allowed the identification of 31 compounds (Table 1) across the four RJ samples using accurate mass of parent ions and corresponding daughter ions from MSMS spectra. Figure 5 shows MSMS spectrum of stachyaose, one of the major marker compounds of RJ sample from Tuban. The UNIFI software also allows automatic interpretation and annotation of MSMS spectrum. The major constituents of the RJs were found to be oligosaccharides, fatty acids, and adenosine monophosphate derivatives. Among these, 10-HDA is the most unique marker constituent that is used to check the quality of the RJ. In this study, the abundance of 10-HDA was found to range from 3% to 4% across the samples. This indicated that all the samples were able to meet the quality criteria set by the International Organization of Standardization and the International Honey Commission. According to these institutions, fresh and genuine RJ should contain a minimum 1.4% of 10-HDA (Flanjak et al., 2017). Meanwhile, the PCA enabled for a comprehensive approach to reveal the distinct chemical differences among the four RJ samples, with the compounds responsible for these differences also identified.

**Table 1:** Chemical markers identified in the royal jelly samples obtained from Tuban (T), Kediri (K), Demak (D), and Banjarnegara (B) of Indonesia.

Component name	Molecular Formula	Estimated Structure	T (% Rel. abundance)	B (% Rel. abundance)	K (% Rel. abundance)	M (% Rel. abundance)
Maltopentaose	C_30_H_52_O_26_		0.81 ± 0.09	N. D	N. D	0.01 ± 0.00
D-Galactonic acid	C_6_H_12_O_7_		14.78 ± 0.27	16.62 ± 0.0.8	16.21 ± 0.33	24.12 ± 0.35
Sucrose	C_12_H_22_O_11_		4.40 ± 0.31	16.47 ± 0.55	18.61 ± 0.48	4.77 ± 0.16
Sebacic acid	C_10_H_18_O_4_		13.66 ± 0.32	11.44 ± 0.26	11.52 ± 0.06	13.23 ± 0.27
4-O-Caffeoylquinic acid	C_16_H_18_O_9_		4.10 ± 0.46	6.78 ± 0.75	4.00 ± 0.23	4.72 ± 0.80
Uridine diphosphate glucose	C_15_H_24_N_2_O_17_P_2_		8.55 ± 0.43	6.78 ± 0.15	7.00 ± 0.25	7.23 ± 0.10
3,10,Dihydroxydecanoic acid	C_10_H_20_O_4_		7.18 ± 0.22	6.02 ± 0.02	6.40 ± 0.08	7.03 ± 0.07
Planteose	C_18_H_32_O_16_		0.52 ± 0.01	3.78 ± 0.40	3.94 ± 0.19	0.68 ± 0.05
10-Hydroxydecanoic acid	C_10_H_20_O_3_		4.85 ± 0.09	3.45 ± 0.34	4.32 ± 0.20	4.47 ± 0.53
Sebacate	C_10_H_16_O_4_		4.02 ± 0.26	3.15 ± 0.08	3.30 ± 0.03	3.91 ± 0.07
10-Hydroxy-2-decenoic acid	C_10_H_18_O_3_		3.79 ± 0.08	2.96 ± 0.44	3.82 ± 0.05	4.03 ± 0.68
(2E)-10-[(10-Hydroxydecanoyl)oxy]-2-decenoic acid	C_20_H_36_O_5_		3.67 ± 0.08	2.90 ± 0.05	3.18 ± 0.10	3.88 ± 0.21
Adenosine monophosphate	C_10_H_14_N_5_O_7_P		2.67 ± 0.08	2.20 ± 0.03	1.69 ± 0.10	2.63 ± 0.15
Adenosine monophosphate derivative	C_10_H_14_N_5_O_7_P	-	2.60 ± 0.21	2.20 ± 0.04	1.73 ± 0.02	2.61 ± 0.20
Isomaltose	C_12_H_22_O_11_		5.45 ± 0.10	2.11 ± 0.03	1.91 ± 0.07	2.83 ± 0.09
Acetoxydecanoic acid derivative	C_12_H_22_O_4_	-	2.62 ± 0.01	2.04 ± 0.02	2.24 ± 0.05	2.14 ± 0.05
Dimethyloctadienoic acid	C_20_H_30_O_5_		0.30 ± 0.01	1.93 ± 0.02	0.72 ± 0.01	0.98 ± 0.02
(2E)-10-[(9-Carboxynonanoyl)oxy]-2-decenoic acid	C_20_H_34_O_6_		2.58 ± 0.10	1.91 ± 0.02	2.10 ± 0.04	2.70 ± 0.04
8,8′-Oxybis(4,4-dimethyloctanoic acid)	C_20_H_38_O_5_		1.68 ± 0.04	1.30 ± 0.02	1.47 ± 0.05	1.82 ± 0.11
3,11-Dihydroxydodecanoic acid	C_12_H_24_O_4_		1.06 ± 0.01	0.85 ± 0.00	0.82 ± 0.02	0.87 ± 0.03
Dihydroxydodecanoic acid derivative	C_12_H_24_O_4_	-	1.05 ± 0.04	0.80 ± 0.01	0.85 ± 0.01	0.91 ± 0.01
Dihydroxydodecanoic acid derivative	C_12_H_24_O_4_	-	1.05 ± 0.03	0.80 ± 0.01	0.85 ± 0.02	0.83 ± 0.01
(2E)-10-Acetoxy-2-decenoic acid	C_12_H_20_O_4_		0.89 ± 0.01	0.70 ± 0.01	0.75 ± 0.02	0.74 ± 0.02
Acetoxydecanoic acid derivative	C_12_H_22_O_4_	-	0.70 ± 0.02	0.65 ± 0.01	0.59 ± 0.01	0.66 ± 0.03
Stachyose	C_24_H_42_O_21_		3.18 ± 0.25	0.58 ± 0.01	0.41 ± 0.04	0.43 ± 0.01
Citric acid	C_6_H_8_O_7_		0.71 ± 0.03	0.58 ± 0.02	0.59 ± 0.03	0.60 ± 0.04
AMP-N-Oxide	C_10_H_14_N_5_O_8_P		0.63 ± 0.04	0.38 ± 0.04	0.35 ± 0.02	0.39 ± 0.01
10-Acetoxydecanoic acid	C_12_H_22_O_4_		0.39 ± 0.01	0.36 ± 0.00	0.33 ± 0.01	0.38 ± 0.00
10-Acetoxy-2-decenoic acid derivative	C_12_H_20_O_4_	-	0.16 ± 0.01	0.14 ± 0.00	0.13 ± 0.00	0.15 ± 0.01
11-Oxododecanoic acid	C_12_H_22_O_3_		0.16 ± 0.01	0.11 ± 0.01	0.13 ± 0.01	0.13 ± 0.01
Verbascose	C_30_H_52_O_26_		1.79 ± 0.12	0.02 ± 0.00	0.02 ± 0.00	0.10 ± 0.00

The analyzed samples all contained fatty acids and oligosaccharides. In fact, the relative abundance of these substances and certain unique chemicals were the only aspects that distinguished them. Here, as Table 1 shows, sample T and sample M were found to be significantly different from the others in terms of containing a greater abundance of organic fatty acids and oligosaccharides. Sample T had the greatest abundance of chemicals such as maltopentaose, sebacic acid, 3,10-dihydroxydecanoic acid, uridine diphosphate glucose, 10-hydroxydecanoic acid, adenosine monophosphate, isomaltose, acetoxydecanoic acid derivative, stachyose, AMP-N-oxide, and verbascose. Among these, the stachyose and verbascose were unique to sample T. Meanwhile, samples B and K had an abundance of sucrose and planteose. Their chemical markers also possessed similar abundance percentages, which were confirmed by their clustering in the PCA scores. Dimethyloctadienoic acid was found to be unique to sample B. The abundance of D-galactonic acid and 10-HDA was found to be highest in sample M, at 24.12 ± 0.35% and 4.03 ± 0.68%, respectively, whereas the abundance of sebacate, (2E)-10-[(10-hydroxydecanoyl)oxy]-2-decenoic acid, adenosine monophosphate derivative, (2E)-10-[(9-carboxynonanoyl)oxy]-2-decenoic acid, and 8,8′-oxybis(4,4-dimethyloctanoic acid) was found to be slightly higher in sample M than that in the other samples. The data also indicated that no chemical markers were identified as unique in either sample K or sample M. The chemical profiling of Indonesian royal jelly has not been very well documented in literature. The most abundant fatty acids of Greek royal jelly were found to be 10-hydroxy-2-decenoic acid, 10-HDA, and sebacic acid (Melliou and Chinou 2005). In comparison with Greek royal jelly, major fatty acids of Inodonesian royal jelly were found to be sebacic acid, 3,10-Dihydroxydecanoic acid, 10-HDA and 10-Hydroxy-2-decenoic acid (Table 1). The reported concentrations of 10-hydroxy-2-decenoic acid and sebacic acid in RJ samples ranges from 3-6% and 0.5% respectively (Zhou et al., 2007 and Moutsatsou et al., 2010). However, in this study sebacic acid was found to be major fatty acid with relative abundance ranging from 11.4 to 13.7% (Table 1) indicating differences in chemical composition of Indonesian RJ versus other types of RJ samples. The relative abundance of 10-hydroxy-2-decenoic acid ranged from 3-4% which is similar to previous reports (Zhou et al., 2007; Moutsatsou et al., 2010).

### 3.3 Chemical profiles of RJ from other geographical origins

By taking into account the dependence of RJ's chemical composition on the geographical origin, the chemical profiles of other RJ harvested from different area should be analyzed as a comparison. Here, we analyzed the chemical profiles of RJ from Italy and Brazil as can be seen in Table 2.

**Table 2:** Chemical Profiles Comparison between Indonesian RJ and other RJs from different geographical origin.

Geographical Origin	Analytical Instrument	Chemical Compounds	References
Indonesia	UPLC-QTOF/MS	*Listed in*Table 1	-
Italy	Nuclear magnetic resonance (NMR)	10-Hydroxy-2-decenoic acid, glucose, fructose, sucrose, proline, lysine, arginine, valine, serine, citric acid, 3-hydroxybutyrric acid, pantothenic acid (VB-5) and compounds with uridinic-, nicotinamidic-, guanosinic- and adenosinic-like structures.	Mazzei et al. (2020)
Brazil	Electrospray ionization mass spectra (ESI) (-) fourier transform ion cyclotron resonance (FT-ICR) mass spectrometry (MS)	8-Hydroxy-octanoic acid, 10-hydroxy-2-decenoic acid, 3-hydroxydecanoic acid, 9,10-dihydroxy-2-decenoic acid, 3,10-dihydroxydecanoic acid, 10-acetoxydecanoic acid and 10,11-dihydroxydodecanoic acid	Schmidt et al. (2015)

Both Italian and Brazilian RJ samples contained 10-hydroxy-2-decenoid acid, which is known as the key component of RJ. Similar to our result, the Italian RJ also contained citric acid and compounds with uridinic- and adenosinic-like structures. The exact structures could not be confirmed due to the low intensity of the NMR signals (Mazzei et al., 2020). On the other side, the Italian RJ also showed the presence of some amino acids, which were not detected in our study. For Brazilian RJ, the sample was mainly consisted of compounds that were also detected in Indonesian RJ, including dihydroxydodecanoic acid derivatives, 10-acetoxydecanoic acid, 3,10-dihydroxydecanoic acid and hydroxydecanoic acid derivative.

### 3.4 Potential of the chemicals for use in cosmetic and skin care products

As this research was aimed at identifying any chemicals that had the potential to be used in cosmetic and bio-supplement products, further examinations had to be conducted. Comparing the identified chemicals with those identified in previous studies in relation to their efficacy for the cosmetic and bio-supplement industries was chosen as the method for the analysis. In terms of cosmetics, a number of chemicals found in RJ have the potential to be used in cosmetic and skin care formulation. Cosmetic compositions must, generally speaking, possess anti-aging properties, skin-moisturizing effects, or skin-whitening properties, in addition to their intrinsic benefits and effects.

RJ is an influential anti-aging product that helps in alleviating the aging process (Sugiyama et al. 2012). Anti-aging properties are known to exist in d-galactonic acid, 10-HDA, and citric acid. D-galactonic acid can be used to alleviate skin wrinkles and capsize the effect of aging on human face skin. Further studies by Honda et al. (2015) and Li et al. (2013) demonstrated that the 10-HDA in RJ has been widely regarded as a healthy substance, one that could slow down the aging process by promoting the normal turnover of skin cells. To be more specific, this compound augments the synthesis of ovulation hormones and maintains a lower expression of the hormones involved in the aging process in young ovarian cells such as the luteinizing and follicle-stimulating hormones (Pasupuleti et al., 2017). Furthermore, tests carried out on the 10-HDA in RJ revealed its moisturizing effect on skin in terms of the stratum corneum improvement after the application of the compound (Gu et al. 2017). In fact, 10-HDA also possesses high antibacterial activities, which are highly beneficial for skin (Yang et al., 2018). Citric acid is an example of an alpha hydroxy acid (AHA) that is renowned for its exfoliant properties. The capabilities of AHA-containing products range from skin moisturizing for reduction of wrinkles to skin exfoliation (Babilas et al. 2012).

Similarly, research on the antimicrobial activity of 3,10-dihydroxydecanoic acid demonstrated that it could inhibit the growth of *Staphylococcus* (*S.*) *epidermidis* at 18/0.25 of the zone of inhibition/minimum inhibitory concentration (mg/mL) (Melliou and Chinou 2005). *S. epidermidis* is the bacteria responsible for the development of acne. RJ's traditional reputation as an antimicrobial agent was confirmed through its inhibitory effect on bacterial growth. Another theory stated that the development of acne vulgaris is triggered by an overgrowth of *Propionibacterium* (*P.*) *acnes*, which can be overcome by the presence of sucrose. Here, Wang et al. (2016) demonstrated that sucrose is able to specifically escalate the probiotic capability of *S. epidermidis* through short-chain fatty acids production that can contribute to *P. acnes* growth suppression.

The skin-whitening effect can be produced by 10-hydroxydecanoic acids and isomaltose, the former of which are unsaturated fatty acids that have similar molecular structures to 10-HDAs. These acids are reported to have a skin-whitening effect in terms of decreasing the melanin synthesis and the tyrosinase activity, which, in turn, results in the downregulation of melanogenesis (Koya-Miyata et al., 2004). In other words, 10-hydroxydecanoic acids can potentially be formulated into a skin-whitening product. Meanwhile, isomaltose is known to possess an antioxidant activity and a relatively high stability, which make it potentially useful as a sunscreen, a skin-refining agent, and a skin-whitening agent.

Finally, the esters of sebacic acid are used as plasticizers in cosmetic products and can act as a pH adjuster for skin (Winter 2009). These medium-chain fatty acids (saturated, unsaturated, and hydroxylated) are reported to possess skin-improving properties. It has also been reported that maltopentaose is used to produce pharmaceuticals and cosmetic products.

### 3.5 Potential of the chemicals for use in bio-supplements

The uses of the identified chemicals are not only restricted to cosmetic and skin care products but also extended to bio-supplement products. In addition to having numerous efficacies for the skin, 10-HDA also has several immunomodulatory properties that make it a suitable constituent of bio-supplements (Sugiyama et al. 2012). Meanwhile, maltopentaose is free of starch odor and has a slight sweetness, meaning it can be used to produce protein diets, and sebacic acid is known to have the anti-inflammatory response (Ahmad et al., 2020) that is found in various supplements. Different properties are represented by 4-O-caffeoylquinic acid, including strong antioxidant activity (Ganzon et al. 2018), meaning it has the potential to be used as a bio-supplement constituent. In addition, the mixture form of isomaltose in conjunction with saccharides such as glucose, maltose, and panose has been widely utilized for food, cosmetics, and medicines.. Research conducted by Li et al. (2013) revealed that stachyose and verbascose can be combined into a consumable prebiotic bio-supplement, which is known as Deshipu stachyose granules (DSG). DSG is composed of 55.3% stachyose and 9.7% verbascose and is regarded as a compound for promoting beneficial intestinal bacteria and inhibiting pathogenic bacteria.

Overall, it can be stated that all the tested samples possessed the required properties for cosmetics and skin care formulation, including anti-aging, antioxidant, acne vulgaris inhibiting, moisturizing, refining, whitening, and exfoliating effects. However, sample T was found to have the highest abundance of fatty acids such as sebacic acid, which indicates that it is perhaps the best among Indonesia's RJs for use in the cosmetics industry. Furthermore, sample T had the greatest potential for use in bio-supplements as it possessed the highest abundance in chemicals that possess antioxidant and prebiotic properties. The existence of large quantities of maltopentaose and isomaltose, which are widely utilized in food ingredients and protein diets, also indicates that the RJs can be transformed into consumable products.

To the best of the authors' knowledge, this is the first study in which the chemical compositions of Indonesian RJ samples were compared in view of better understanding the differences among them. In fact, in this study, we focused on largely profiling the lipid compounds, and further work is required to identify and profile other minor compounds such as amino acids or vitamins to obtain a more complete picture of the chemical composition of Indonesian RJs.

## 4 Conclusions

The application of chemical profiling by using UPLC-QTOF/MS in terms of four types of RJ from different locations in Indonesia was carried out for the first time and resulted in the identification of >30 compounds. The multivariate statistical analysis was successful in distinguishing between the four types of species. The results of this study also indicated that the fatty acids and the related compounds of RJ could be responsible for its skin-protective properties such as anti-aging, moisturizing, skin-whitening, skin-refining, and pH-adjusting effects. In addition, RJ has the potential to improve body functioning through pharmacological activities such as antioxidant, anti-inflammatory, and immunomodulatory effects. All the analyzed samples possessed these skin-protective and body-function-improvement properties as they all contain fatty acids and oligosaccharides. The abundance percentages and certain unique chemicals were all that distinguished them. According to the results, it was clear that the sample from Demak (M) had good potential for use in the cosmetics and skin care industries. However, the higher abundance of fatty acids and oligosaccharides in the RJ from Tuban (T) indicated that it perhaps has the highest potential among Indonesia's RJs for these fields. Sample T also had more potential to be used in bio-supplements as it possessed the highest abundance in chemicals that possess antioxidant, anti-inflammatory, and prebiotic properties. These statements are based on comparing the chemicals with those used in previous research, and further clinical trials should be carried out in the near future to confirm the efficacy of each RJ for skin improvement and the extent to which these RJs can support the body's internal functioning as part of a bio-supplement. The finding has led to a conclusion that our non-targeted approach with an integrated and rapid workflow demonstrated good potential for the valuation of complex natural products such as RJs and their utilization in commercial products especially cosmetic and bio – supplement.

## Declarations

### Author contribution statement

Eka Sari: Conceived and designed the experiments; Performed the experiments; Analyzed and interpreted the data. Wrote the paper.

Kaysa Faradis Mahira: Analyzed and interpreted the data; Wrote the paper.

Dhavalkumar Narendrabhai Patel: Analyzed and interpreted the data; Contributed reagents, materials, analysis tools or data.

Lee Suan Chua, Diah Kartika Pratami: Analyzed and interpreted the data.

Muhamad Sahlan: Performed the experiments; Analyzed and interpreted the data.

### Funding statement

This work was supported by The Directorate of Research and Community Service (DRPM) 10.13039/501100006378Universitas Indonesia through Grant Publikasi Terindeks Internasional (PUTI) Kolaborasi Internasional (2Q2) (No: NKB785/UN2.RST/HKP.05.00/2020).

### Data availability statement

Data included in article/supplementary material/referenced in article.

### Declaration of interests statement

The authors declare no conflict of interest.

### Additional information

No additional information is available for this paper.
